# Differentiated Contributions of Executive Functions to Domain-Specific Numerical Precursors in Preschool Children

**DOI:** 10.3390/jintelligence14100253

**Published:** 2026-10-10

**Authors:** Davide Apicerni, Paolo Stievano, Giulia Brandi, Lilian Zotti, Sergio Melogno, Lina Pezzuti

**Affiliations:** 1 Department of Dynamic and Clinical Psychology and Health Studies, Sapienza University of Rome, Via degli Apuli, 1, 00185 Rome, Italy; paolo.stievano@uniroma1.it (P.S.); giulia.brandi@uniroma1.it (G.B.); lilian.zotti@uniroma1.it (L.Z.); lina.pezzuti@uniroma1.it (L.P.); 2 ASL ROMA 2, Via Monza, 2, 00182 Rome, Italy; 3 Faculty of Psychology, “Niccolò Cusano” University of Rome, Via Don Carlo Gnocchi, 3, 00166 Rome, Italy; sergio.melogno@unicusano.it

**Keywords:** executive functions, working memory, inhibitory control, cardinality, approximate number system, rapid automatized naming, preschool mathematics, digital assessment, DAP-T

## Abstract

Executive functions (EFs) are well-established predictors of early mathematical development, yet whether distinct components contribute differentially to specific numerical precursors during the preschool years remains unclear. The present study examined the unique contributions of inhibitory control, verbal short-term memory (VSTM), visuospatial working memory (VSWM), and set-shifting to three precursors (procedural cardinality, non-symbolic quantity reproduction, and Approximate Number System [ANS] performance) while controlling for the domain-general contributions of receptive language and rapid automatized naming (RAN) and tested whether age moderated these associations. A total of 316 Italian preschoolers (M = 59.85 months, range 38–76; 158 females) completed nine tasks from the DAP-T tablet-based battery; hierarchical regression and moderation analyses were conducted on multiply imputed datasets (50 imputations), with Holm–Bonferroni correction applied to individual coefficients and complete-case sensitivity analyses performed to assess robustness. Age moderation was examined through six interaction terms with Bonferroni correction. Age was the strongest predictor across outcomes. Above and beyond age and domain-general covariates, procedural cardinality was uniquely predicted by VSTM (β = 0.205); ANS performance by inhibitory control (β = 0.191) and RAN (β = 0.252); non-symbolic quantity reproduction by RAN (β = 0.182) and age alone. RAN emerged as the most consistent cross-domain predictor of numerical performance after age, with effects surviving correction on non-symbolic quantity reproduction and ANS. Set-shifting contributed to no outcome robustly after correction. None of the six age-moderation interactions survived Bonferroni correction; a single nominal effect emerged for Age × VSTM on procedural cardinality (*p* = .044), which should be regarded as exploratory. EFs thus show a differentiated profile across numerical precursors, with VSTM and inhibitory control as primary executive supports for procedural cardinality and non-symbolic comparison, respectively, whereas non-symbolic quantity reproduction appears to draw more on domain-general processing efficiency than on standard executive control.

## 1. Introduction

Academic learning prerequisites constitute a set of foundational competencies that underlie the development of domain-specific school-based skills, including reading, writing, arithmetic, comprehension, and fine motor abilities (Usai et al., 2007). These cognitive precursors, which are acquired during the first five years of life, play a critical role in the subsequent acquisition and automatization of formal academic learning (Whitehurst & Lonigan, 1998). They are broadly classified into two categories: domain-general prerequisites, referring to general cognitive abilities that transversally predict performance across academic subjects, and domain-specific prerequisites, which are more narrowly tied to particular learning domains (Passolunghi & Lanfranchi, 2012). The concept of prerequisites rests on a hierarchical and constructive view of learning, according to which the acquisition of a given competency is both temporally and functionally contingent on the prior mastery of another. The analysis of pre-learning supports the early identification of children at risk for learning difficulties (Tretti et al., 2002) and can be framed within the broader construct of school readiness, which encompasses the set of cognitive, linguistic, socio-emotional, and behavioral competencies that enable children to adapt successfully to the demands of formal schooling (Duncan et al., 2007). Within this framework, executive functions (EF) and early numerical competencies are widely recognized as key indicators of readiness for later academic achievement.

### 1.1. Domain-General and Domain-Specific Precursors of Numerical Competence

In the years preceding primary school entry, a range of cognitive abilities serve as critical precursors of academic learning, functioning as early indicators of future skill development across core scholastic domains. Among these, certain abilities operate transversally, being implicated in both literacy and mathematical development. The domain-general competencies enable individuals to acquire, process, and deploy new information in the service of complex cognitive tasks, and constitute the foundational substrate upon which learning processes are built (De Vita et al., 2018). They encompass attention and executive functions (EFs; Miyake et al., 2000; Diamond, 2013), general intellectual ability, expressive and receptive language, motor and visuomotor skills, rapid automatized naming, visuospatial competencies, and working memory. The transversal nature of these abilities contributes to the construction of the cognitive infrastructure required for academic success (Koponen et al., 2013; Passolunghi & Lanfranchi, 2012; Hjetland et al., 2017; Peng & Fuchs, 2016; Usai et al., 2018; ISS, 2022).

During the preschool period, the development of mathematical abilities rests on a set of domain-specific competencies directly linked to numerical cognition. According to the Triple Code Model (Dehaene, 1992; Dehaene & Cohen, 1996; Dehaene et al., 2025; Skagenholt et al., 2021), numerical representation engages three distinct systems (visual-symbolic, verbal, and analogue magnitude) such that early numerical development necessarily draws on both symbolic and non-symbolic abilities (Raghubar & Barnes, 2017).

On the non-symbolic side, a central role is played by the Approximate Number System (ANS), a biologically grounded system present from early developmental stages that enables the perception, comparison, and manipulation of numerical magnitudes in an approximate, non-symbolic manner, without recourse to counting or symbolic strategies (Raghubar & Barnes, 2017). The ANS is conceptualized as an unbounded mental number line along which quantities are represented in spatial order, with acuity that sharpens progressively over the course of development (Östergren & Träff, 2013; Kolkman et al., 2013). Non-symbolic numerical abilities also include the capacity to perform transformations on physical quantities, such as adding or removing elements from concrete or depicted sets, constituting a form of informal arithmetic that precedes the acquisition of conventional symbolic notation and predicts subsequent performance in symbolic calculation (Kolkman et al., 2013). Although the predictive contribution of ANS acuity to formal mathematical competence remains debated, converging evidence suggests that it contributes to the development of early arithmetic skills, particularly in comparison and estimation tasks, and that its effect is partly mediated by the acquisition of symbolic representations (De Smedt et al., 2013; Mazzocco et al., 2011; Vallortigara & Panciera, 2014).

On the symbolic side, the relevant competencies include mastery of the verbal count sequence from rote recitation of number words to cardinal counting that maps each number word onto individual elements of a set, and understanding of the cardinality principle, the insight that the last number word produced represents the total numerosity of the set (Gelman & Gallistel, 1978). These procedural components are complemented by the recognition and naming of Arabic numerals, symbolic magnitude comparison, abilities that together constitute a critical bridge between informal numerical cognition and formal mathematical thinking (Merkley & Ansari, 2016; Vanbinst & De Smedt, 2016). Symbolic competencies acquired during the preschool period and counting skills, in particular, are among the most robust and direct predictors of subsequent mathematical achievement (Raghubar & Barnes, 2017; Mera et al., 2026), whereas domain-general processes primarily serve a supportive function in the acquisition and consolidation of these abilities.

### 1.2. Executive Functions and Mathematical Abilities

Among domain-general skills, the literature agrees in attributing to EFs a pervasively relevant role in the development of academic skills. They constitute a set of high-level cognitive processes responsible for the control, regulation, and planning of goal-directed behavior and reasoning (De Vita et al., 2018). Among the most influential theoretical frameworks, the model proposed by Miyake et al. (2000) identifies three core components whose development is observable from the preschool period onward: inhibitory control, working memory, and set-shifting, conceptualized as distinct yet interdependent domains. Inhibitory control refers to the ability to suppress automatic or irrelevant responses, supporting the selection of task-relevant information and sustaining selective attention (Posner & DiGirolamo, 1998). Working memory is a capacity-limited short-term storage system that enables the temporary retention, elaboration, and active manipulation of verbal and visuospatial information during the execution of cognitive tasks (Baddeley, 2010). Set-shifting (named also cognitive flexibility) allows individuals to modify strategies and response patterns in accordance with changing task demands, drawing the coordinated engagement of inhibitory control and working memory to disengage from previously established schemas and adopt new solutions (Diamond, 2013). During the preschool years, these elements follow a layered path of growth: working memory appears and stabilizes first, then inhibitory control strengthens quickly near age 3, while cognitive flexibility emerges later, drawing on those two base systems (Shokrkon & Nicoladis, 2022).

A substantial body of research has documented significant associations between EFs and academic achievement, with particular relevance to the mathematical domain (Diamond, 2013). Meta-analytic evidence indicates moderate but reliable associations between EF components and numerical competencies, with broadly comparable effect sizes across the three domains (Emslander & Scherer, 2022; Zhong et al., 2022). At a functional level, each component contributes differentially: inhibitory control is implicated in the suppression of impulsive responses and has been associated with arithmetic and problem-solving performance even after controlling for cognitive and linguistic variables (Clark et al., 2010; Espy et al., 2004; Harvey & Miller, 2017); working memory (particularly its visuospatial component) supports the maintenance and manipulation of numerical information, and shows strong associations with both early numeracy skills and later mathematical achievement (Bull et al., 2008; Viterbori et al., 2015; De Vita et al., 2021); cognitive flexibility seems more closely tied to conceptual math, including numerical relations and switching between strategies (Purpura et al., 2016; Bernal-Ruiz et al., 2023). Beyond visual and spatial skills, verbal working memory, particularly the phonological loop, adds independent support to early number skills because cardinal counting demands temporary storage and order of number words (Baddeley, 2010; Raghubar & Barnes, 2017; Shvartsman & Shaul, 2024). This link is not fixed: longitudinal data show that verbal working memory’s influence shifts during preschool, with mutual effects appearing later (Viesel-Nordmeyer et al., 2022), suggesting age may shape the working memory–math connection.

Beyond the differential contributions of individual EF components, the involvement of EFs in early mathematical development also varies as a function of the type of numerical task. Specifically, EFs show stronger and more consistent associations with symbolic numerical competencies (such as counting and cardinality) than with non-symbolic tasks involving approximate quantity processing, with their predictive weight increasing as tasks require greater reliance on deliberate, controlled processing. This pattern aligns with cross-sectional evidence showing that working memory and inhibitory control contribute to multiple components of preschool mathematical ability, including counting, quantity comparison, and logical reasoning, even after controlling for age and vocabulary (Espy et al., 2004; Harvey & Miller, 2017; Montoya et al., 2018). It also converges with longitudinal findings that preschool executive functions predict later mathematical achievement above and beyond general intellectual ability and language skills (Bull et al., 2008; Clark et al., 2010; Viterbori et al., 2015).

### 1.3. Domain-General Cognitive Covariates: Receptive Language and Rapid Automatized Naming

Beyond EFs and domain-specific numerical precursors, two additional cognitive skills may shape early numeracy across areas: receptive language and rapid automatized naming (RAN). Adding these controls offers a theoretically grounded way to account for linguistic and processing differences that might otherwise be linked to executive functioning.

Receptive language represents an important cross-domain resource for early numerical development. Within the Pathways to Mathematics model (LeFevre et al., 2010), linguistic abilities, particularly receptive vocabulary, constitute one of the pathways through which children acquire early mathematical knowledge, alongside quantitative and spatial-attentional processes. Language supports numerical learning by providing the representational and communicative framework through which children acquire number words and counting sequences, understand quantitative relations, and establish mappings between symbolic number forms and their verbal labels. Consistent with this account, early language abilities predict specific numeracy skills even after nonverbal cognitive abilities are taken into account (Purpura et al., 2011). Vocabulary knowledge has also been associated with foundational preschool competencies such as verbal counting, one-to-one correspondence, and cardinality knowledge, with patterns of association partly distinct from those observed for working memory (Purpura & Ganley, 2014). Receptive language is therefore relevant both as a developmental resource for constructing verbally mediated numerical representations and as a potential source of variance in performance on verbally administered numerical tasks. In the present study, partialling out receptive language consequently provides a more stringent test of the unique contribution of executive functions to early numerical precursors, reducing the possibility that associations attributed to executive processes instead reflect individual differences in verbal comprehension or access to linguistically mediated numerical knowledge.

RAN has been investigated primarily as a predictor of reading fluency (McWeeny et al., 2022), but accumulating evidence indicates that its predictive relevance extends to numerical and arithmetic development. Rather than indexing a single cognitive ability, RAN is a multicomponent task that recruits phonological, lexical, visuo-perceptual, attentional, and serial-processing mechanisms (Agostini et al., 2022). Its relevance across domains may therefore reflect the efficiency with which familiar visual stimuli are rapidly identified, accessed, mapped onto verbal or symbolic representations, and retrieved in sequence. These mechanisms are particularly relevant to skills that increasingly depend on fluent and automatized retrieval, including reading, counting, and arithmetic fact retrieval (Koponen et al., 2013). Consistent with this interpretation, RAN predicts arithmetic performance beyond phonological awareness and verbal short-term memory (Koponen et al., 2013), while broader factors such as processing speed, working memory, and attention may partly account for its association with achievement across academic domains (Georgiou et al., 2013). In the absence of a standardized intelligence measure administered to the full sample, RAN was included in the present study as a proxy for efficiency in rapid access to and automatized retrieval of familiar symbolic representations. Controlling for this source of variability provides a more conservative test of whether executive functions explain unique variance in domain-specific numerical precursors beyond processing demands shared across cognitive and academic tasks.

### 1.4. The Present Study

Drawing on converging evidence identifying EFs as a critical support system for early mathematical development and highlighting their differential contributions to distinct aspects of numerical cognition, the present study aimed to investigate the predictive role of executive function components with respect to specific domain-specific numerical precursors in preschool-aged children, namely cardinal counting, non-symbolic quantity reproduction, and non-symbolic quantity comparison (ANS). Given the theoretical relevance of receptive language and RAN as domain-general covariates of early numerical development, these two abilities were included as control variables in all analyses, in the absence of a standardized measure of general intelligence for the full sample. First, it was hypothesized that working memory (both verbal and visuospatial), inhibitory control, and cognitive flexibility would be significantly associated with early numerical skills, consistent with evidence documenting reliable associations between EF components and numerical competence in the preschool years (Emslander & Scherer, 2022; Zhong et al., 2022; Purpura et al., 2016) (Hypothesis 1). Second, it was predicted that the three EF components would contribute differentially to distinct dimensions of numerical competence, including counting and quantity discrimination, in line with evidence that executive functions are more strongly associated with symbolic than with non-symbolic numerical tasks and that distinct components support distinct numerical skills (Coolen et al., 2021; Wongupparaj & Kadosh, 2022; Simanowski & Krajewski, 2019) (Hypothesis 2). Finally, given evidence that working memory (both verbal and visuospatial) is among the most relevant executive predictors of early numerical skills (Bull et al., 2008; Purpura et al., 2016; Viterbori et al., 2015) and that its relationship with numerical development may change over the course of the preschool years (Viesel-Nordmeyer et al., 2022), we explored whether age moderated the association between working memory components (verbal and visuospatial) and the three numerical precursors (Research Question 1). Given the mixed evidence on age-related change in the working memory–mathematics relationship, this analysis was conducted without a specific directional hypothesis and is best interpreted as exploratory.

## 2. Materials and Methods

### 2.1. Study Design

The present study adopted a cross-sectional, observational design aimed at examining the differential contributions of executive function components to domain-specific numerical precursors in preschool-age children. Data were collected as part of a broader research protocol focused on the validation and normative development of the Developmental Assessment for Preschoolers Tool (DAP-T), a tablet-based neuropsychological battery for early learning precursors.

### 2.2. Participants

The study was carried out in compliance with the principles outlined in the Declaration of Helsinki and received approval from the Ethics Committee of Sapienza University of Rome (protocol No. 249/2024; approved on 16 December 2024). A convenience sampling strategy was adopted, based on pre-existing research collaborations between the university department and preschool institutions. Participants were recruited from three preschool institutions in central Italy and two preschool institutions located in northern Italy that had previously established research collaborations with the university department. Recruitment was preceded by two informational meetings (one with teachers and one with parents and/or legal guardians) aimed at presenting the study objectives and procedures. Written informed consent was obtained from parents or legal guardians for all participating children. Participation was voluntary, and families were informed of their right to withdraw at any time without consequences; no identifying information was included in the dataset used for analyses. The consent form also collected basic sociodemographic information, including the primary language spoken at home and the presence of any prior neurodevelopmental diagnoses. All children whose families provided consent participated in the assessment phase, with no exclusions prior to data collection. For the purposes of data analysis, however, children who were not native Italian speakers or who had received diagnoses of developmental disorders, neurological conditions, or other clinical conditions potentially affecting task performance were excluded.

A total of 326 Italian-speaking children recruited from the five preschool institutions completed the administered tasks. Following the application of the inclusion and exclusion criteria, ten participants were excluded from the analytical sample: seven due to a diagnosis of a neurological disorder and three due to non-native Italian language background. The final sample comprised 316 children (158 females, 50.0%), ranging in age from 38 to 76 months (M = 59.85, SD = 9.66), enrolled across the three years of preschool education. Demographic characteristics of the sample are reported in Table 1.

### 2.3. Procedures

All assessment activities were carried out in a dedicated room within the school, using a one-to-one administration format. Data collection was conducted by the first author of the study together with trained psychology students. Before data collection began, the psychology students took part in a series of training sessions conducted by the first author, which included a theoretical presentation of the tasks and their administration procedures, followed by peer-to-peer practice. During the data collection phase, the first author was present on site at every assessment session in the participating schools, ensuring adherence to a standardized administration protocol. Prior to the beginning of the assessment, verbal assent was obtained from each child, with procedures tailored to their level of verbal comprehension, in order to minimize potential discomfort and ensure a voluntary and supportive participation context. Task administration followed a fixed sequence integrated into the tablet-based application: Magic Box, Sentence Repetition, Morphosyntactic Comprehension, Animal House, Rapid Automatized Naming, What’s in Common, Approximate Number System, Golden Tokens, and Ball Jar. The sequence was designed to alternate cognitively demanding tasks with less demanding ones in order to minimize the impact of fatigue and to preserve engagement across the session. The sequence was designed to distribute cognitively demanding tasks across the session in order to minimize the impact of fatigue and to preserve engagement in the preschool-aged sample. Each child completed a single administration session, which lasted approximately 30 min on average.

### 2.4. Measures

The present study is part of a broader research program aimed at the validation of the DAP-T assessment battery, a digitalized tool designed to measure domain-general and domain-specific precursors of literacy and mathematical learning in preschool-aged children (Apicerni et al., 2026). The DAP-T is a web-based application accessible through an online platform and compatible with multiple operating systems, including iOS, Android, Windows, and macOS. The software delivers all task stimuli, records touchscreen interactions, and stores raw data in formats suitable for subsequent statistical analyses. It was developed by an external provider under the supervision of the research team. At the time of writing, the DAP-T is available exclusively in Italian for research purposes. Researchers interested in employing the instrument within collaborative projects may contact the corresponding author for further information regarding access conditions.

For the purposes of the present study, performance was analyzed on a subset of battery tasks assessing executive domain [inhibitory control, visuospatial working memory (VSWM), verbal short-term memory (VSTM)], early numeracy skills [non-symbolic quantity comparison (ANS), non-symbolic quantity reproduction, and cardinal counting], receptive language [morphosyntactic comprehension] and rapid automatized naming [RAN].

1. Magic Box Task (Inhibitory Control). This task, adapted from the Pinball task described in Carlson’s (2016) review, consists of 8 items and requires the child to hold down a red button displayed on the screen until an auditory go-signal is presented. Waiting intervals increase progressively across items. A gift box displayed at the center of the screen opens to provide positive feedback when an item is passed correctly and remains closed to signal an incorrect response. The software records touchscreen responses and assigns one point for each correctly completed item, as well as reaction time in milliseconds. The task yields an accuracy score (maximum = 8) and provides a measure of response inhibition. Internal consistency, estimated via McDonald’s Omega, was ω = 0.767. Concurrent validity was supported by a significant correlation with the FEPS 2–6 Stroop task (Usai et al., 2017), adopted as a reference measure of inhibitory control (ρ = 0.250, *p* = .020).
2. Sentence Repetition Task (VSTM). This task comprises six pre-recorded sentences: two consisting of five words, two of ten words, and two of fifteen words. Sentences were presented one at a time via the tablet’s audio output, and the child was asked to repeat each sentence aloud immediately after hearing it. Three performance indices were derived. First, an overall accuracy score (maximum = 12) was computed based on criteria independent of phonetic or phonological errors: two points were assigned for correct verbatim repetition, one point when all target words were present but produced in a different order without intrusions, and zero points when not all target words were reproduced. Second, a lexical unit index was calculated, corresponding to the total number of target words correctly repeated across all sentences, regardless of order and independently of the accuracy score (maximum = 60). Third, a word span index was derived, defined as the maximum sentence length (in number of words) repeated correctly (maximum = 15). To relate the performance in this test with that in the other tests, the accuracy score was considered. Internal consistency, estimated via McDonald’s Omega, was ω = 0.829. Convergent evidence with Sentence Repetition subtest of the BVL 4–12 battery (Marini et al., 2015), used as the reference measure of verbal short-term memory, confirmed the task’s concurrent validity (ρ = 0.677, *p* < .001).
3. Animal House Task (Visuospatial Short-Term Memory). This task, adapted from the Visual Patterns Test (Della Sala et al., 1997), consists of 9 items and assesses visuospatial memory through the presentation of stimulus configurations within grids of increasing size: 2 × 2 (four cells, two stimuli), 3 × 3 (nine cells, four stimuli), and 4 × 4 (sixteen cells, eight stimuli). Stimuli appear simultaneously and remain visible for a duration proportional to their numerosity (one second per stimulus; e.g., four seconds in the 3 × 3 grid). The child is required to observe the positions of the stimuli and, once they disappear, to indicate their previous locations via touchscreen. Responses are recorded automatically by the software. The task yields an accuracy score (maximum = 42) and a visuospatial pre-span parameter, defined as the number of correct responses prior to the first error. The task primarily assesses the storage component of visuospatial short-term memory, as well as visuospatial working memory updating processes. To compare the performance in this test with that in the other tests, the accuracy score was taken into account. Internal consistency, estimated via McDonald’s Omega, was ω = 0.780. The task showed a significant positive association with the Corsi Blocks Test (Pezzuti et al., in preparation), used as the reference measure of visuospatial short-term memory (ρ = 0.357, *p* < .001).
4. What’s in Common Task (Set-Shifting). This task is based on an adaptation of the Something’s the Same (STS) Game developed by Zelazo, as described in the review by Willoughby et al. (2012). In each item, two figures sharing a common feature (shape, color, or size) are initially displayed in the upper portion of the screen within a rectangular frame. Shapes may be circles, triangles, or stars; colors may be green, orange, or blue; sizes may be large or small. A third figure subsequently appears in the lower portion of the screen, separated from the first two, and shares one specific feature with one of the upper figures. The possible feature pairings are Color & Color-Size; Size & Size-Color; Shape & Shape-Size; Size & Size-Shape; Color & Color-Size; and Shape & Shape-Color. The child is asked to indicate which of the two upper figures shares a feature with the lower one. Correct responses are followed by positive auditory feedback, and incorrect responses by a negative sound. The software records touchscreen responses and reaction times, computing accuracy and speed scores. The task measures the ability to flexibly redirect attentional focus (attentional shifting). To compare the performance in this test with that in the other tests, we considered the accuracy score. Internal consistency, estimated via McDonald’s Omega, was ω = 0.790. Given the absence of a directly comparable set-shifting measure in the reference battery, concurrent validity was examined against two theoretically related constructs that the executive-function literature identifies as core components underlying the task’s demands (Diamond, 2013; Miyake et al., 2000): processing speed, indexed by the WPPSI-III Symbol Search subtest (Wechsler Preschool and Primary Scale of Intelligence, Third Edition; Wechsler, Italian edition, Sannio Fancello & Cianchetti, 2008), and visuospatial short-term memory, indexed by the Corsi Blocks Test (Pezzuti et al., in preparation). Significant positive correlations emerged with both criteria (Symbol Search: ρ = 0.345, *p* = .002; Corsi Blocks: ρ = 0.387, *p* < .001), supporting the construct-level validity of the task.
5. Golden Tokens Game (Non-Symbolic Quantity Reproduction). The child observes an animation on the tablet screen in which a given number of tokens (range: 3–7) are dropped one by one into a piggy bank and is subsequently asked to reproduce the same quantity in a virtual piggy bank by dragging tokens individually across the screen with a finger. The task consists of 6 items; the software automatically records responses and computes an accuracy score (range: 0–1), used as an index of non-symbolic quantity representation ability. Internal consistency, estimated via McDonald’s Omega, was ω = 0.772. Concurrent validity was evaluated using two related measures: the BIN 4–6 Non-Symbolic Quantity Comparison subtest (Molin et al., 2006), which reflects early quantitative skill, and the BVN 5–11 Counting subtest (Bisiacchi et al., 2005), which reflects counting-based numerical processing. Significant positive associations appeared with both benchmarks (BIN: ρ = 0.495, *p* < .001; BVN Counting: ρ = 0.438, *p* < .001), indicating that the task shows concurrent validity across non-symbolic and counting elements of early numerical ability.
6. Approximate Number System Task—ANS (Non-Symbolic Quantity Comparison). This task involves the simultaneous and tachistoscopic presentation (2000 ms) of two sets of black dots displayed within two rectangular frames. The task consists of 10 items; numerosities were designed in accordance with Weber’s law, such that discrimination is easier as the ratio between the two quantities increases and more difficult as it decreases (Feigenson et al., 2004). The child is asked to indicate, via touchscreen, the frame containing more dots (Phase 1, items 1–5) or fewer dots (Phase 2, items 6–10). The software records both response accuracy (range: 0–1) and response time, providing a measure of Approximate Number System functioning. Internal consistency, estimated via McDonald’s Omega, was ω = 0.713. Concurrent validity was documented through a significant relationship with the BIN Non-Symbolic Quantity Comparison subtest (Molin et al., 2006), used as the reference measure of the same construct (ρ = 0.539, *p* < .001).
7. Ball Jar Game (Cardinal Counting; procedural application of the cardinal principle). This task, adapted from the Give-N paradigm (Coolen et al., 2021), was designed to assess children’s ability to apply the cardinal principle through a procedural counting task. The cardinal principle (the understanding that the last number word in a count sequence denotes the total quantity of the set) requires the integration of several sub-competencies: knowledge of the forward count sequence, one-to-one correspondence between number words and counted items, tracking of counted and uncounted elements, and application of the stop rule to terminate counting at the target quantity (Gelman & Gallistel, 1978; Sarnecka & Carey, 2008). In the task, the child hears a target numerosity (range: 2–10) produced by the software and is required to place the corresponding number of balls into a virtual container by dragging them across the screen with a finger. The task consists of 12 items and yields an accuracy score (maximum = 12), used in the present study as an index of procedural cardinal counting ability. Internal consistency, estimated via McDonald’s Omega, was ω = 0.966. Its concurrent validity was corroborated by a significant correlation with the BVN 5-11 Counting subtest (Bisiacchi et al., 2005), used as the reference measure of counting ability (ρ = 0.432, *p* < .001).
8. Morphosyntactic Comprehension Task (Receptive Language). This task assesses the child’s ability to understand syntactically complex sentences. The child listens to a pre-recorded sentence and must identify the target referent among several visual options displayed on the screen. The task comprises nine sentences involving three syntactic structures: relative, dative, and negative clauses. Responses are collected via touchscreen and recorded automatically by the software. The task yields an accuracy score, computed by assigning one point for each correct response (maximum = 9), used in the present study as an index of morphosyntactic comprehension and lexical access. Internal consistency, estimated via McDonald’s Omega, was ω = 0.580. A significant association with the BVN 5–11 Syntactic Comprehension subtest (Bisiacchi et al., 2005), used as the reference measure of receptive syntactic skills, supported the task’s concurrent validity (ρ = 0.488, *p* < .001).
9. Rapid Automatized Naming Task (RAN). This task, adapted from the classic RAN paradigm (Denckla & Rudel, 1976), assesses the speed and accuracy of lexical retrieval. The child is required to name, as quickly and accurately as possible, the pictures displayed in a matrix, whose lexical labels are two-syllable words (“Palla”, “Ball”; “Casa”, “House”). Vocal responses are recorded via the tablet’s microphone. The task yields an accuracy score, defined as the total number of pictures correctly named within a 60 s time limit (maximum = 80), used as an index of rapid lexical access and processing speed. Internal consistency, estimated via McDonald’s Omega, was ω = 0.970. Concurrent validity was supported by a significant correlation with the RIAS-2 rapid naming task (Pezzuti et al., 2021), adopted as the reference measure of rapid lexical retrieval (ρ = 0.590, *p* < .001).

### 2.5. Data Analysis

An a priori power analysis was conducted during the study-planning phase, before data collection, using G*Power 3.1 (Faul et al., 2009), within the broader research protocol underlying the DAP-T validation study. The protocol specified an expected effect size of ρ = 0.25 for bivariate associations and f^2^ = 0.15 (a medium effect according to Cohen’s conventions) for the incremental contribution of predictor blocks in multiple regression analyses, with α = 0.05 and power (1 − β) = 0.80. The resulting protocol-defined minimum sample sizes were 149 and 146 participants, respectively, and the final sample (N = 316) exceeded both thresholds.

A post hoc sensitivity power analysis was then conducted to determine the minimum effect sizes detectable with the available sample size (N = 316) at 80% power. With α = 0.05, the study was adequately powered to detect bivariate associations of r ≥ 0.16, an overall multiple-regression effect of f^2^ ≥ 0.046, and incremental effects in the hierarchical regression models ranging from f^2^ = 0.025 to 0.031, depending on the number of predictors added at each step. For the moderation analyses, after Bonferroni correction for the six interaction tests (α = 0.0083), the minimum detectable effect for each individual interaction term was f^2^ = 0.039. For gender comparisons, assuming two equally sized groups (*n* = 158 each), the minimum detectable standardized mean difference was d = 0.32.

Given these thresholds, the study was adequately powered to detect small effects across all planned analyses, indicating that null findings, particularly for set-shifting contributions and age-moderation effects, cannot be attributed to insufficient statistical power.

To test the study hypotheses, a series of planned statistical analyses were conducted. First, descriptive statistics were computed for all study variables, and multivariate outliers were identified using Mahalanobis distance (Tabachnick & Fidell, 2013). As the number of subjects who completed the administered tests is not homogeneous, the pattern of missing data was examined via Little’s MCAR test and bias-reduced logistic regression models, which indicated that missingness was systematically related to observed variables (see the Missing Data Analysis and Multiple Imputation subsection Section 3.3).

Because data collection covered schools across several Italian regions, potential geographic variation was examined across nine cognitive and numerical measures. Schools were grouped into two broad areas by location (Central versus Northern Italy). For each measure, a linear regression model used geographic area as the predictor and age in months as a covariate, with heteroskedasticity-consistent (HC1) standard errors. Models were fitted on 50 imputed datasets and merged using Rubin’s rules, applying Holm–Bonferroni correction across the nine outcomes. A complete-case sensitivity analysis followed the same method. Missing data were addressed through multiple imputation by chained equations (MICE) using the mice package in R, producing 50 imputed datasets with 20 iterations per chain and predictive mean matching for continuous variables. All later analyses used the imputed datasets, combining parameter estimates and standard errors via Rubin’s rules; complete-case replications served as sensitivity checks.

Bivariate correlations were then computed on the imputed datasets to examine associations among EF components (inhibitory control, VSTM, VSWM, and set-shifting) and domain-specific numerical precursors (procedural cardinality, non-symbolic quantity reproduction, and non-symbolic quantity comparison) using methods appropriate to the distributional properties of the data, as determined by normality testing. Effect sizes were interpreted according to Cohen’s (2013) criteria: small (r = 0.10), medium (r = 0.30), and large (r = 0.50). Gender differences across all cognitive measures were examined using linear models with robust HC1 standard errors, with *p*-values corrected via Holm’s procedure to control for multiple comparisons.

To assess the unique and differential contributions of EF components to each numerical outcome, three separate five-step hierarchical linear regression models were estimated, one for each dependent variable. Age in months was entered as a covariate in Step 1 to control for developmental variability. Receptive language and RAN were added in Step 2 as background cognitive covariates. VSTM and VSWM were entered simultaneously in Step 3, inhibitory control was included in Step 4, and set-shifting was entered in Step 5. This stepwise structure was theoretically motivated, reflecting the relative developmental precedence and empirical salience of each executive component as documented in the literature (Diamond, 2013; Miyake et al., 2000; Shokrkon & Nicoladis, 2022). For each model, the incremental variance explained (ΔR^2^) through D1 comparisons between pooled nested models, and regression assumptions, including normality of residuals, absence of multicollinearity (assessed via VIF and tolerance statistics), and independence of errors (assessed via the Durbin–Watson statistic), were verified prior to interpretation. To address the multiplicity of tests across the three models, Holm–Bonferroni correction was applied to individual predictor *p*-values within each model.

Hierarchical regressions were additionally replicated on complete-case samples as sensitivity analyses; results were interpreted as robust when convergent across the MI and complete-case analyses.

Finally, to test the hypothesis that age moderates the relationship between working memory and early numerical skills, moderation analyses were conducted by including interaction terms between VSTM, VSWM, and age in months, respectively. This focus on working memory was theoretically motivated: among EF components, working memory (and VSWM in particular) has been most consistently associated with numerical abilities and later mathematical achievement in the literature (Bull et al., 2008; Purpura et al., 2016; Viterbori et al., 2015; De Vita et al., 2021). These analyses were intended to examine whether the predictive strength of working memory components varied as a function of children’s developmental level within the preschool age range considered. All continuous predictors were mean centered within each imputed dataset, robust HC1 standard errors were used, and Bonferroni correction (adjusted α = 0.0083) was applied to the six interaction terms tested across the three moderation models.

All statistical analyses were conducted in R version 4.5.3 (R Core Team, 2026). Missing-data patterns were examined using the naniar package (Tierney & Cook, 2023). Multiple imputation by chained equations, pooling of regression estimates, and pooled model comparisons were performed using mice (van Buuren & Groothuis-Oudshoorn, 2011), while pooled Spearman correlations across imputed datasets were obtained using *miceadds* (Robitzsch & Grund, 2026). Bias-reduced logistic regression models used to examine predictors of missingness were estimated with brglm2 (Kosmidis et al., 2020), with Type II Wald tests obtained using *car* (Fox & Weisberg, 2019). Heteroskedasticity-robust HC1 standard errors for the moderation and gender analyses were estimated using *estimatr* (Coppock et al., 2026). Complete-case sensitivity analyses were conducted using base R functions. Internal consistency coefficients (McDonald’s Omega) for the DAP-T tasks were computed using jamovi version 2.6 (The jamovi project, 2024).

## 3. Results

### 3.1. Descriptive Statistics

Preliminary descriptive analyses were conducted to examine the distributional properties of all study variables. As reported in Table 2, the majority of variables showed significant departures from normality, as indicated by Shapiro–Wilk tests. Non-parametric statistical methods were therefore adopted for subsequent bivariate analyses.

### 3.2. Multivariate Outlier Analysis

Multivariate outliers were examined using Mahalanobis distance computed on the variables included in the regression models (Tabachnick & Fidell, 2013). A conservative threshold based on the chi-square distribution (df = 9, *p* < .001; χ^2^ = χ^2^ = 29.588) was adopted. The maximum observed Mahalanobis distance (*D*^2^ = 31.48) slightly exceeded this cutoff, indicating the presence of two potential multivariate outliers. These cases were further inspected and deemed substantively plausible, with no evidence of data entry errors or anomalous response patterns. Accordingly, the observations were retained in the final dataset. Given the relatively large sample size and the well-documented robustness of regression analyses to minor violations of distributional assumptions, the retention of this case was not expected to substantially bias the results.

### 3.3. Missing Data Analysis and Multiple Imputation

Missing data rates varied from 1.6% (receptive vocabulary) to 15.2% (RAN), with the highest rates observed for RAN (n = 48; 15.2%), procedural cardinality (n = 42; 13.3%), non-symbolic quantity reproduction (n = 32; 10.1%), and ANS (n = 23; 7.3%). Age, gender, school, and class year were completely observed. Little’s MCAR test rejected the missing-completely-at-random assumption, χ^2^(39) = 71.577, *p* = .001. Bias-reduced logistic regressions revealed that missingness in the three numerical outcomes was systematically associated with class year (procedural cardinality: *p* = .002; non-symbolic quantity reproduction: *p* = .002; ANS: *p* = .027), and for RAN and non-symbolic reproduction, also with school (both *p* ≤ .020). Gender showed no association with missingness on any outcome. Descriptively, missing rates were predominantly found among younger children (e.g., RAN missingness decreased from 27.5% in first-year to 5.2% in third-year preschoolers), aligning with a missing-at-random mechanism conditional on observed variables.

Missing data were managed through multiple imputation by chained equations (MICE; van Buuren & Groothuis-Oudshoorn, 2011) implemented in R, generating 50 imputed datasets with 20 iterations per chain and predictive mean matching for continuous variables. The imputation model incorporated all analysis variables along with age, gender, school, class year, and passive terms for age × VSTM and age × VSWM interactions to ensure compatibility with the moderation analyses. Convergence diagnostics indicated adequate mixing without systematic trends, and imputed values adhered to the observed ranges of all scales and replicated the developmental gradients across class years.

Multiple imputation served as the primary analytic approach, providing unbiased estimates under the missing-at-random assumption. Complete-case replications of the hierarchical regressions were conducted as sensitivity analyses, employing listwise deletion on all model variables to retain only participants with complete data on the outcome and on all seven predictors of the model.

### 3.4. Gender Differences

To characterize the sample in terms of gender balance and to determine whether gender should be controlled for in the subsequent analyses, independent-samples comparisons were conducted on the nine cognitive and numerical measures. Comparisons were performed on the imputed datasets through linear models with robust HC1 standard errors, coding gender as 0 = females and 1 = males. To account for the multiplicity of tests, the nine *p*-values were corrected via Holm’s procedure. After correction, no significant gender differences emerged on any of the measures examined. Before correction, two nominal effects had been observed: males scored lower than females on VSTM (B = −0.515, SE = 0.239, *p* = .032, 95% CI [−0.986, −0.044]) and on set-shifting (B = −0.821, SE = 0.334, *p* = .015, 95% CI [−1.479, −0.164]). Both effects fell below the significance threshold after Holm correction (p_Holm = 0.258 and p_Holm = 0.131, respectively). No nominal differences were observed for inhibitory control, VSWM, RAN, receptive language, procedural cardinality, non-symbolic quantity reproduction, or ANS. Given the absence of significant gender differences after correction, gender was not included as a covariate in the subsequent regression and moderation models.

### 3.5. Geographic Area Differences

Of the 316 participants, 236 (74.7%) were recruited from schools in central Italy and 80 (25.3%) from schools in northern Italy. After adjustment for age and Holm-Bonferroni correction across the nine outcomes, geographic area was significantly associated only with inhibitory control (B = 1.06, SE = 0.26, β = 0.46, p_Holm < 0.001), with children in northern Italy showing higher scores than those in central Italy. No significant differences emerged for the remaining eight outcomes (all p_Holm ≥ 0.540). The complete-case sensitivity analysis confirmed this pattern, with the inhibitory control effect showing an identical standardized magnitude across the two approaches (β_MI = β_CC = 0.46). Because geographic area was defined at the school level, this result should be interpreted as a group-level association rather than as evidence of a causal effect of geographic origin.

### 3.6. Bivariate Correlations

Pooled Spearman correlations among age, executive function components, background cognitive covariates, and numerical outcomes were computed on the imputed datasets (Table 3). All associations with the three numerical outcomes were positive and statistically significant at uncorrected pooled tests.

Age showed the strongest associations with the three numerical outcomes (procedural cardinality ρ = 0.578; non-symbolic quantity reproduction ρ = 0.542; ANS ρ = 0.493), consistent with the substantial developmental changes occurring across the preschool period.

Among the executive components, VSWM and VSTM correlated most strongly with procedural cardinality (ρ = 0.445 and 0.433, respectively), and both showed moderate associations with the other two outcomes (VSWM: ρ = 0.387 with non-symbolic quantity reproduction, ρ = 0.343 with ANS; VSTM: ρ = 0.335 with non-symbolic quantity reproduction, ρ = 0.334 with ANS). Set-shifting was most strongly associated with non-symbolic quantity reproduction (ρ = 0.365), while showing weaker correlations with procedural cardinality (ρ = 0.250) and ANS (ρ = 0.256). Inhibitory control showed the weakest associations across the executive components (ρ = 0.208–0.292 across outcomes).

Among the background cognitive covariates, RAN emerged as a consistent correlate of all three outcomes (procedural cardinality ρ = 0.477; non-symbolic quantity reproduction ρ = 0.466; ANS ρ = 0.443), showing the strongest associations after age. Receptive language (syntactic comprehension) showed moderate correlations with all outcomes (procedural cardinality ρ = 0.357; ANS ρ = 0.280; non-symbolic quantity reproduction ρ = 0.249). All the executive components and covariates were also positively correlated with age (range ρ = 0.266–0.581), with the strongest associations for VSWM (ρ = 0.581) and RAN (ρ = 0.561), and the weakest for inhibitory control (ρ = 0.266).

### 3.7. Hierarchical Regression Models

Three hierarchical regression models were estimated on the imputed datasets, each addressing one numerical outcome. Predictors were added in five steps guided by theory: age (Step 1), receptive language and RAN (Step 2), VSTM and VSWM (Step 3), inhibitory control (Step 4), and set-shifting (Step 5). This sequence followed a developmental-precedence logic, placing earlier-differentiating executive components before later ones, in line with research on executive function structure in preschool children (Karr et al., 2018; Simanowski & Krajewski, 2019). Coefficients were pooled using Rubin’s rules, and incremental variance was tested with D1 comparisons between nested models. To manage multiple testing, Holm–Bonferroni correction was applied to individual predictor *p*-values within each model (seven predictors per model). Standardized regression coefficients (β) were calculated to compare predictor strength across models. The results of the three hierarchical regression models appear in Table 4.

Procedural cardinality. Age explained 34.3% of the variance in Step 1. Step 2 (receptive language + RAN) yielded a significant increment, ΔR^2^ = 0.044, F(2, 250.63) = 8.14, *p* < .001, as did Step 3 (working memory components), ΔR^2^ = 0.044, F(2, 266.01) = 9.28, *p* < .001. Inhibitory control (Step 4) produced a marginal increment, ΔR^2^ = 0.011, F(1, 160.32) = 3.74, *p* = .055, while set-shifting (Step 5) did not contribute significantly, ΔR^2^ = 0.001, *p* = .594. The final model accounted for 44.3% of the variance. Significant unique predictors were age (B = 0.132, SE = 0.028, β = 0.317, *p* < .001, 95% CI [0.077, 0.186]), VSTM (B = 0.388, SE = 0.109, β = 0.205, *p* < .001, 95% CI [0.172, 0.603]), and RAN (B = 0.047, SE = 0.018, β = 0.169, *p* = .011, 95% CI [0.011, 0.083]). VSWM did not reach significance (β = 0.094, *p* = .102), inhibitory control was borderline (β = 0.109, *p* = .054), and receptive language and set-shifting were non-significant (both *p* ≥ .594). After Holm–Bonferroni correction, age and VSTM survived correction (age: p_Holm < 0.001; VSTM: p_Holm = 0.003); the RAN effect fell above the corrected threshold (p_Holm = 0.055).

Non-symbolic quantity reproduction. Age explained 29.3% of the variance in Step 1. Step 2 yielded a significant increment, ΔR^2^ = 0.033, F(2, 284.17) = 6.35, *p* = .002. The working memory block (Step 3) and inhibitory control (Step 4) did not contribute significantly (ΔR^2^ = 0.008, *p* = .245; ΔR^2^ = 0.002, *p* = .468, respectively). Set-shifting (Step 5) produced a small but nominally significant increment, ΔR^2^ = 0.012, F(1, 282.49) = 4.90, *p* = .028. The final model accounted for 34.9% of the variance. Significant unique predictors were age (B = 0.074, SE = 0.015, β = 0.360, *p* < .001), RAN (B = 0.025, SE = 0.009, β = 0.182, *p* = .006), and set-shifting (B = 0.080, SE = 0.036, β = 0.118, *p* = .028). Receptive language, VSTM, VSWM, and inhibitory control were non-significant (all *p* ≥ .190), with VSWM in particular showing a substantially attenuated coefficient in the present model (β = 0.048, *p* = .437). After Holm–Bonferroni correction, age (p_Holm < 0.001) and RAN (p_Holm = 0.037) survived, while the set-shifting effect did not (p_Holm = 0.138).

ANS. Age explained 22.4% of the variance in Step 1. Step 2 yielded a significant increment, ΔR^2^ = 0.062, F(2, 243.17) = 9.41, *p* < .001, and Step 3 produced a smaller but significant increment, ΔR^2^ = 0.018, F(2, 280.71) = 3.13, *p* = .045. Inhibitory control (Step 4) yielded a further significant increment, ΔR^2^ = 0.033, F(1, 212.64) = 11.47, *p* < .001; set-shifting (Step 5) was non-significant, ΔR^2^ = 0.001, *p* = .586. The final model accounted for 33.7% of the variance. Significant unique predictors were inhibitory control (B = 0.150, SE = 0.044, β = 0.191, *p* < .001, 95% CI [0.062, 0.238]), RAN (B = 0.031, SE = 0.009, β = 0.252, *p* = .001, 95% CI [0.013, 0.050]), and age (B = 0.041, SE = 0.013, β = 0.219, *p* = .002, 95% CI [0.015, 0.067]). VSTM approached significance (β = 0.115, *p* = .062); receptive language, VSWM, and set-shifting were non-significant (all *p* ≥ .413). After Holm–Bonferroni correction, all three predictors survived (inhibitory control: p_Holm = 0.006; RAN: p_Holm = 0.007; age: p_Holm = 0.010).

Across the three models, Holm–Bonferroni correction supported the stability of the main results: age consistently predicted all outcomes, while different executive factors aligned with each numerical skill (VSTM for procedural cardinality and inhibitory control for ANS). RAN acted as a general predictor of numerical performance, showing strong effects on ANS and non-symbolic quantity reproduction, plus a marginal effect on procedural cardinality. Set-shifting provided no unique contributions that survived correction in any model. Standardized coefficients indicated that age was the strongest single predictor for procedural cardinality (β = 0.317) and non-symbolic quantity reproduction (β = 0.360), whereas RAN led for ANS (β = 0.252); the executive predictors that remained significant (VSTM on cardinality: β = 0.205; inhibitory control on ANS: β = 0.191) had effect sizes similar to those of the domain-general covariates.

### 3.8. Moderation Analyses

To test whether age moderated the relationship between working memory components and numerical performance, six moderation terms were examined across three general linear models, one per numerical outcome. Each model included age, VSTM, and VSWM as main effects, along with the two-way interactions between age and each working memory component. All continuous predictors were mean-centered within each imputed dataset. Robust HC1 standard errors were used to accommodate potential departures from homoscedasticity. Given the six interaction terms tested across the three models, Bonferroni correction was applied (adjusted α = 0.0083).

None of the six interaction terms survived Bonferroni correction (Table 5). At the uncorrected threshold, only the age × VSTM interaction on procedural cardinality reached significance (B = −0.017, SE = 0.008, *p* = .044, 95% CI [−0.033, −0.001]), while this effect did not survive correction (p_Bonferroni = 0.262). The negative sign of the coefficient indicated that the VSTM–cardinality association tended to attenuate with age, that is, VSTM was more strongly associated with procedural cardinality in younger preschoolers, with this association becoming weaker in older children. No other interaction approached significance for procedural cardinality (age × VSWM: *p* = .448), non-symbolic quantity reproduction (age × VSTM: *p* = .318; age × VSWM: *p* = .490), or ANS (age × VSTM: *p* = .350; age × VSWM: *p* = .504).

Overall, these results did not provide robust support for the hypothesis that age moderates the relationship between working memory components and numerical performance. The single nominally significant interaction, involving VSTM and procedural cardinality, is reported for completeness but should be interpreted cautiously in light of the correction for multiple testing.

### 3.9. Sensitivity Analysis: Complete-Case Replication

To assess how well the main results hold up under possible violations of the missing-at-random assumption, the three hierarchical regression models were repeated using complete-case samples. Each outcome used a separate subset of participants with full data on that outcome and all seven predictors (procedural cardinality N = 223; non-symbolic quantity reproduction N = 234; ANS N = 238). Because Little’s test had already ruled out the MCAR assumption, these estimates should not be viewed as unbiased; instead, they acted as sensitivity checks to see which effects remained stable across methods. The findings appear in Table 6.

The main findings were largely confirmed, both in statistical significance and in the magnitude of standardized coefficients. For procedural cardinality, the incremental contribution of the working memory block remained significant in the complete-case analysis (ΔR^2^ = 0.030, *p* = .007), and VSTM emerged as a significant unique predictor in the final model (B = 0.336, SE = 0.110, β = 0.186, *p* = .003), closely matching the MI estimate (β_MI = 0.205). Age and RAN also retained significant effects (age: B = 0.137, β = 0.354, *p* < .001; RAN: B = 0.046, β = 0.175, *p* = .008), again with coefficients highly consistent across the two approaches (age: β_MI = 0.317, β_CC = 0.354; RAN: β_MI = 0.169, β_CC = 0.175).

For ANS, the incremental contribution of inhibitory control was confirmed (ΔR^2^ = 0.021, *p* = .009), and inhibitory control emerged as a significant unique predictor in the final model (B = 0.105, SE = 0.040, β = 0.152, *p* = .009). RAN retained a robust effect (B = 0.026, β = 0.246, *p* < .001), as did age (B = 0.051, β = 0.321, *p* < .001). The standardized coefficients confirmed the pattern observed in the MI analysis, with RAN emerging as the strongest predictor of ANS performance in both approaches (β_MI = 0.252; β_CC = 0.246), followed by age (β_MI = 0.219; β_CC = 0.321) and inhibitory control (β_MI = 0.191; β_CC = 0.152).

For non-symbolic quantity reproduction, age (B = 0.078, β = 0.371, *p* < .001) and RAN (B = 0.027, β = 0.185, *p* = .007) remained significant unique predictors, with near-identical standardized magnitudes across the two approaches (age: β_MI = 0.360, β_CC = 0.371; RAN: β_MI = 0.182, β_CC = 0.185).

Two effects that emerged in the MI analyses were not confirmed in the complete-case analyses. The incremental contribution of the working memory block to ANS was not confirmed (MI: ΔR^2^ = 0.018, *p* = .045; CC: ΔR^2^ = 0.005, *p* = .476). Similarly, the incremental contribution of set-shifting to non-symbolic quantity reproduction, nominally significant in the MI analysis (ΔR^2^ = 0.012, *p* = .028), was not confirmed in the complete-case analysis (ΔR^2^ = 0.008, *p* = .112); the set-shifting coefficient in the final complete-case model was positive but non-significant (B = 0.064, SE = 0.040, β = 0.096, *p* = .112). The magnitude of this effect on reproduction was moderately attenuated across the two approaches (β_MI = 0.118; β_CC = 0.096), a pattern consistent with a fragile finding rather than with a substantially different underlying effect. These divergences, together with the failure of these effects to survive Holm-Bonferroni correction in the MI analyses, indicate that they should be interpreted with caution and await replication.

Overall, the convergence between the MI (primary) and complete-case (sensitivity) analyses supported the robustness of the pattern documented in the main analyses: VSTM as a distinctive predictor of procedural cardinality, inhibitory control as a distinctive predictor of ANS performance, and RAN as a consistent predictor of numerical performance across the three outcomes. Standardized coefficients for the four effects surviving Holm-Bonferroni correction in the MI analyses showed absolute differences between MI and CC estimates below |Δβ| = 0.10 across all comparisons, further supporting the robustness of these findings.

## 4. Discussion

The present study examined the differential contributions of four EF components to three domain-specific numerical precursors in preschool-aged children: procedural cardinality, non-symbolic quantity reproduction, and non-symbolic quantity comparison (ANS), while controlling for the domain-general contributions of receptive language and rapid automatized naming (RAN). The findings support a differentiated profile of EFs involvement across distinct dimensions of early numerical cognition, consistent with theoretical and empirical accounts positing that the relationship between EFs and mathematics is not uniform but varies systematically as a function of the specific numerical skill under consideration.

The correlational analyses indicated that the three domain-specific numerical precursors were significantly associated with all executive components and with both domain-general covariates though with markedly different magnitudes across predictors and outcomes (Cohen, 2013). Working memory components consistently produced medium-sized associations with the three numerical outcomes, with VSWM showing slightly stronger correlations than VSTM. Inhibitory control showed small-to-medium correlations that were modest yet consistent across all outcomes. Set-shifting generated small-to-medium associations, with the strongest link appearing in non-symbolic quantity reproduction and similar coefficients for the remaining outcomes. Among domain-general factors, RAN stood out as the strongest single correlate of the three numerical precursors, producing medium-sized associations that matched or surpassed any executive predictor. Receptive language showed small-to-medium associations, weaker than those of RAN but consistent across outcomes. Two patterns are worth noting at this preliminary stage. First, the magnitude of executive-numerical associations was broadly comparable across the three outcomes, with no single component showing markedly stronger correlations than the others; this suggests that a differentiated profile of executive involvement, if present, would emerge more clearly once shared variance with age and domain-general processes is partialled out. Second, the strength of RAN correlations, exceeding those of most executive components, foreshadows a substantial domain-general contribution to early numerical development that any test of specifically executive effects should take into account.

Examining in greater detail the role of the individual executive components in early numerical skills, the hierarchical regressions showed that, across all three models, age in months emerged as the strongest predictor of numerical performance, accounting for 34.3%, 29.3%, and 22.4% of the variance in procedural cardinality, non-symbolic quantity reproduction, and ANS performance, respectively. This finding reflects the rapid and broad growth of numerical competencies that characterizes the preschool period (Esplin et al., 2021; Litkowski et al., 2020). Of particular interest, however, is the decreasing gradient of variance explained by age, which was highest for procedural cardinality and lowest for ANS. This pattern suggests that symbolic numerical abilities may follow a steeper developmental trajectory within the preschool range than non-symbolic quantity processing, plausibly reflecting the greater sensitivity of symbolic skills to instructional exposure and to the progressive mastery of the count sequence. This interpretation converges with evidence that the development of symbolic number knowledge accelerates markedly once children grasp the cardinality principle, at which point growth in symbolic skills comes to outpace that of non-symbolic abilities (Geary, 2018). It is further consistent with findings that the contribution of symbolic and mapping-based numerical performance to overall mathematics increases following the onset of formal instruction, whereas non-symbolic quantity processing shows a comparatively stable developmental course across the preschool-to-primary transition (Hischa et al., 2025).

Beyond age, the pattern of executive contributions differed markedly across numerical outcomes, supporting the hypothesis of a differentiated rather than uniform relationship between executive components and early numerical competencies (Hypothesis 2). This differentiation is consistent with a growing body of evidence indicating that EFs are more strongly and consistently associated with symbolic than with non-symbolic numerical skills: a latent executive factor robustly predicts symbolic mathematics and its growth in 3- and 4-year-olds (Coolen et al., 2021), and EFs make a unique contribution specifically to symbolic skills, suggesting that these require additional cognitive control and are dissociable from non-symbolic processing when domain-general abilities are modeled simultaneously (Ashkenazi & Adi, 2023). In line with this view, multivariate models of early mathematical development position EFs (and VSTM in particular) among the core mechanisms supporting the manipulation of numerical information, whereas non-symbolic abilities assume a more marginal role once symbolic skills are controlled for (Mera et al., 2026). In the following sections, we first consider the transversal role of RAN as a domain-general predictor, and then examine the specific configuration of executive predictors for each of the three numerical outcomes.

Beyond age and executive factors, RAN stood out as a strong and steady predictor of early numerical skill. It added a meaningful independent effect to both non-symbolic quantity reproduction and ANS, holding after Holm–Bonferroni correction and remaining in the complete-case sensitivity analysis; procedural cardinality showed a similar nominal effect but failed to meet correction for multiple comparisons. This positions RAN as the most reliable cross-domain predictor among the three numerical precursors after age. In theoretical terms, the pattern aligns with the idea that RAN reflects cognitive processes supporting automatic retrieval across academic domains, capturing the efficient learning and recall of arbitrary links between visual symbols and phonological codes, the same processes recruited by fluent reading, counting, and arithmetic fact retrieval (Koponen et al., 2013). Consistent with this account, longitudinal evidence indicates that the RAN-mathematics link is largely unidirectional (Georgiou et al., 2020), and that shared domain-general processes such as processing speed and attention partially explain its cross-domain predictive value (Georgiou et al., 2013). In the present study, the transversal contribution of RAN indicates that early numerical development is supported not only by executive components but also by the efficiency with which children rapidly access and retrieve symbolic-verbal representations. This provides a partial control for domain-general processing efficiency in the interpretation of the executive contributions reported below.

Procedural cardinality was uniquely predicted by VSTM after controlling for age and domain-general covariates, with the effect surviving Holm–Bonferroni correction. Inhibitory control also contributed to the model at the block level, but its individual coefficient did not survive correction for multiple comparisons and should therefore be interpreted with caution. The contribution of VSTM is consistent with theoretical accounts emphasizing the role of phonological and verbal resources in sustaining the mental representation of the count sequence, mapping number words onto quantities, and maintaining intermediate results during counting procedures (Baddeley, 2010; De Vita et al., 2021; Purpura et al., 2016). Indeed, the nature of the task, which is based on the Give-N paradigm, requires the child to hold the target numerosity in memory while carrying out the counting procedure. Notably, VSTM emerged as the sole executive predictor of procedural cardinality despite the fact that its bivariate correlation with the outcome was numerically comparable to that of VSWM. This pattern is consistent with a scenario in which VSWM shares substantial variance with age and RAN, so that its bivariate association is absorbed once these predictors are partialled out, whereas the verbal-specific contribution of VSTM to the counting procedure remains unique. Taken together, these findings are consistent with recent evidence from three- and four-year-olds indicating that EFs support the counting and enumeration processes that, in turn, enable the conceptual acquisition of cardinality, a construct rooted more directly in counting and enumeration than in EFs per se (Morosini et al., 2025). Within this account, executive resources may operate primarily by scaffolding the procedural skills through which cardinality is established, rather than by directly supporting cardinal knowledge. These findings extend prior work documenting the specific contribution of verbal working memory to early symbolic mathematical skills (Bull et al., 2008; Viterbori et al., 2015) and underscore the verbal-specific nature of executive support for symbolic numerical processing.

ANS performance was predicted by age, RAN, and inhibitory control, whereas neither working memory component reached significance, a more selective profile than that observed for procedural cardinality. This finding converges with a substantial literature on the role of inhibitory control in non-symbolic quantity comparison. Dot-comparison tasks systematically pit numerical magnitude against continuous perceptual features such as cumulative surface area, density, and item size, requiring children to suppress these salient but numerically irrelevant cues in order to access numerosity, a process that depends on inhibitory control, particularly in incongruent trials where perceptual and numerical information conflict (Guo & Wei, 2024; Rodríguez & Ferreira, 2023). Developmental work further indicates that the influence of non-numerical perceptual features decreases over development and that younger children are especially susceptible to perceptual interference in these tasks (Starr et al., 2017; Szűcs et al., 2013). That inhibitory control, but not working memory, uniquely predicted ANS performance is consistent with the view that the central demand of non-symbolic comparison lies in interference suppression rather than in the storage and manipulation of information. The independent contribution of RAN, discussed above, further supports this account by indicating that ANS performance also draws on domain-general processing efficiency, over and above executive control. The literature is not unanimous on this point, however, as some studies report that ANS acuity predicts early mathematics independently of inhibitory control (Keller & Libertus, 2015; Starr et al., 2017), indicating that the relative roles of numerical acuity and interference suppression remain a matter of debate.

Non-symbolic quantity reproduction was largely accounted for by age (β = 0.360) and by RAN (β = 0.182), both of which survived Holm–Bonferroni correction and were confirmed in the complete-case sensitivity analysis. None of the four executive components emerged as a robust predictor of reproduction performance after correction for multiple comparisons. Set-shifting yielded a nominal positive effect (β = 0.118, *p* = .028) that did not survive Holm–Bonferroni correction (p_Holm = 0.138) and was not replicated in the complete-case sensitivity analysis (*p* = .112); this contribution should therefore be regarded as tentative and interpreted with caution. Notably, VSWM, which had emerged as a modest predictor of quantity reproduction in earlier analyses without domain-general covariates, showed a substantially attenuated coefficient in the present model (β = 0.048, *p* = .437). This is consistent with a scenario in which its bivariate association with reproduction is largely absorbed by the variance shared with age and RAN once these are partialled out. The pattern illustrates the importance of controlling for domain-general processing efficiency when evaluating executive contributions to non-symbolic numerical skills.

The relative independence of non-symbolic quantity reproduction from standard executive components is theoretically interpretable. Indeed, the task involves the sequential presentation of stimuli, one at a time, a format that, as shown by studies sharply distinguishing simultaneous from sequential presentation, appears to rely on a specific system for the temporal processing of stimuli (Anobile et al., 2019). The absence of symbolic elements, such as naming the numerosity or counting aloud, may explain why VSTM was not a significant predictor in this task, in contrast to procedural cardinality. Accordingly, the counting ability required here may depend more heavily on domain-specific processes related to quantity recognition, as well as on basic attentional processes involved in tracking sets of objects (X. Zhang, 2016; Trick et al., 2003). The inclusion of such measures represents a useful direction for future research. Within this account, RAN’s substantial contribution, comparable to the strongest executive predictors for the other two outcomes, suggests how efficiently children track and retrieve quantity representations. This process relies on domain-general processing speed and attentional resources rather than deliberate cognitive control. The nominal association with set-shifting, if replicated in future work, might reflect the demands of flexibly shifting attention between the target quantity and the child’s own reproduction attempt; the present data, however, do not warrant strong claims in this direction.

The dissociation between VSTM and VSWM deserves particular consideration. VSTM predicted procedural cardinality but not the other outcomes, indicating domain-specific rather than domain-general involvement. This verbal specificity is somewhat at odds with a prominent strand of the literature emphasizing the visuospatial component as a particularly robust predictor of early mathematics (Bull et al., 2008; Viterbori et al., 2015). However, it converges with longitudinal evidence that the phonological loop becomes the working memory component most closely linked to numerical development across the preschool years, while the visuospatial sketchpad contributes only indirectly through reciprocal relations with VSTM and the central executive (Viesel-Nordmeyer et al., 2022). A plausible interpretation is that the relative contributions of the two working memory components depend on the specific numerical skill assessed: procedural cardinality, with its strong reliance on the verbal count sequence, may preferentially engage phonological resources, whereas VSWM may be more relevant for spatially demanding or magnitude-based tasks. This account is consistent with findings that distinct working memory components support different numerical skills depending on their representational demands, with counting-based mechanisms uniquely predicting symbolic rather than non-symbolic processing (Finke et al., 2021).

Set-shifting did not contribute significantly to any of the three outcomes in the regression models, after correction for multiple comparisons. It did, however, yield a nominal effect on non-symbolic quantity reproduction, which did not survive Holm–Bonferroni correction and was not replicated in the complete-case sensitivity analysis. This nominal contribution should therefore be regarded as tentative and in need of replication. More broadly, set-shifting showed significant bivariate correlations with all three numerical outcomes, but these associations did not translate into robust unique contributions once age, domain-general covariates, and the other executive components were entered into the regression models. A plausible interpretation lies in the developmental structure of EFs at this age: confirmatory factor analytic work has shown that, in 5-year-olds, updating already forms a distinct factor, whereas inhibition and shifting converge onto a single, very highly correlated factor, indicating that these two components are not yet functionally dissociable in the preschool years (Simanowski & Krajewski, 2019). At this developmental stage, shifting may therefore be too closely intertwined with inhibitory control to account for additional variance once inhibition is included in the model, consistent with the broader view that cognitive flexibility is the latest-developing executive component (Buttelmann & Karbach, 2017).

The robustness of the reported findings was evaluated through a complete-case sensitivity analysis, in which the hierarchical regression models were re-estimated on the subset of participants with no missing data on any variable included in the models. Across the three outcomes, the pattern of standardized coefficients was highly consistent between the multiple-imputation and complete-case analyses, supporting the interpretation that the substantive conclusions of the study do not depend on the missing-data-handling procedure. Age emerged as the strongest predictor in both approaches for all three outcomes (β_MI = 0.317–0.360; β_CC = 0.354–0.371), with RAN as the second most consistent predictor across models (β_MI = 0.169–0.252; β_CC = 0.175–0.246). Similarly, the two effects that survived Holm-Bonferroni correction in the multiple-imputation analysis were confirmed in the complete-case analysis: VSTM on procedural cardinality (β_MI = 0.205; β_CC = 0.186) and inhibitory control on ANS (β_MI = 0.191; β_CC = 0.152). A different pattern emerged for two nominal findings. First, the contribution of set-shifting to non-symbolic quantity reproduction was significant at the individual-coefficient level in the multiple-imputation analysis (β_MI = 0.118, *p* = .028) but did not survive Holm–Bonferroni correction (p_Holm = 0.138) and was not replicated in the complete-case analysis (β_CC = 0.096, *p* = .112). Second, the contribution of inhibitory control to procedural cardinality was borderline in both analyses (p_MI = 0.054; p_CC = 0.113) and did not survive correction in either. These two effects should therefore be regarded as tentative and in need of replication. Overall, the convergence between multiple-imputation and complete-case results strengthens confidence in the four robust findings (age and RAN as transversal predictors, VSTM as the executive predictor of procedural cardinality, and inhibitory control as the executive predictor of ANS), while identifying the specific effects that call for further empirical scrutiny.

Regarding the exploratory analysis of age moderation (Research Question 1), none of the six interaction effects tested survived Bonferroni correction for multiple comparisons (α = 0.0083). A single nominal effect emerged for the Age × VSTM interaction on procedural cardinality (B = −0.017, SE = 0.008, 95% CI [−0.033, −0.001], *p* = .044, p_Bonf = 0.262), with a negative direction indicating that the association between VSTM and procedural cardinality was stronger in younger children and weakened as age increased. This finding did not reach the level of statistical robustness required to survive correction and should therefore be regarded as exploratory and preliminary. Notwithstanding these caveats, the observed pattern is theoretically interpretable and converges with evidence indicating that the association between verbal working memory and numerical performance declines with age, whereas the contribution of spatial working memory remains comparatively stable across development (Y. Zhang et al., 2022). A plausible developmental account is that the cognitive processes underlying procedural cardinality shift over the preschool years. Younger children, whose counting is still effortful and not yet automatized, may rely more heavily on verbal working memory to maintain the target numerosity and the count sequence, which renders VSTM particularly relevant for them. With age, however, counting procedures become increasingly automatic (Girelli et al., 2000), plausibly reducing the demand on verbally mediated resources. Taken together, this pattern suggests that the involvement of VSTM in cardinal counting may peak early and decline as procedural skills consolidate. This interpretation remains post hoc, and the moderation effect (which did not survive correction and was confined to a single component–outcome combination) requires replication with longitudinal designs and process-level measures of counting strategy use before more definitive conclusions can be drawn.

## 5. Limitations

Several limitations of the present study should be acknowledged. First, the cross-sectional design precludes causal inference regarding the direction of the observed relationships and does not allow determination of whether the executive profiles identified here predict subsequent mathematical trajectories. Longitudinal designs across the preschool-to-primary transition would be needed to address this question.

Second, no standardized assessment of general intelligence was given to the entire sample. To address this gap, RAN served as a proxy for broad processing efficiency, while receptive language acted as a control for verbal comprehension. Although this dual control aligns with theories that treat these factors as central to numerical growth, it cannot replace a direct measure of fluid intelligence. Future research should ideally add a standardized IQ test alongside RAN and receptive language to better control general cognitive ability.

Third, each EF component was indexed by a single task indicator, which precludes a formal latent-variable test of the structural dissociability of executive components, most notably for the inhibition–shifting distinction discussed earlier. Future work employing multiple indicators per component and a latent-variable approach could be needed to formally estimate the dissociability of EFs in relation to early numerical skills.

Fourth, the sample was recruited through convenience sampling from three preschool institutions in central Italy, which limits geographical and sociodemographic generalizability. Most critically, socioeconomic status (SES) was not assessed and could not be controlled for in the analyses. Evidence indicates that SES predicts both EF development and early mathematical competence, and that inhibitory control in particular mediates this association in preschool children (Rémeau et al., 2026). The omission of SES therefore could represent a potential confound, and future studies should include sociodemographic covariates to partition the unique contributions of EFs from the broader environmental context.

Fifth, the ANS task did not include a systematic manipulation of congruence between numerical magnitude and continuous perceptual features (e.g., cumulative surface area, density, item size). This limits the precision with which the inhibitory control contribution to ANS performance can be attributed specifically to interference suppression, as opposed to more general demands of the comparison procedure (Gilmore et al., 2014). Future studies employing congruence-controlled ANS paradigms would allow a more targeted test of this mechanism.

Sixth, the numerical precursors assessed represent only a subset of the competencies relevant to early mathematical development. The study did not include measures of symbolic magnitude comparison, number line estimation, subitizing, or symbolic arithmetic. Their associations with executive components, which differ from those documented here, have been reported elsewhere and would enrich the picture provided by the present findings.

Finally, the exploratory analysis of age moderation yielded a single nominal effect (Age × VSTM on procedural cardinality) that did not survive Bonferroni correction and was not systematically tested in a longitudinal framework. Any developmental interpretation of this finding therefore remains provisional and requires replication with longitudinal designs and process-level measures of counting strategy use.

## 6. Conclusions

The present study examined the differential contributions of four executive function components (inhibitory control, verbal short-term memory, visuospatial working memory, and set-shifting) to three domain-specific numerical precursors in preschool-aged children, while controlling for the domain-general contributions of receptive language and rapid automatized naming. Four findings emerged as robust across multiple-imputation and complete-case analyses. First, age accounted for the largest share of variance across all three numerical outcomes, with a decreasing gradient from procedural cardinality to non-symbolic quantity comparison. Second, verbal short-term memory was the sole executive predictor of procedural cardinality, consistent with the verbal demands of the counting procedure. Third, inhibitory control uniquely predicted non-symbolic quantity comparison, in line with its role in suppressing perceptual interference during dot-comparison tasks. Fourth, rapid automatized naming proved to be the most consistent cross-domain predictor of numerical performance, supporting its role as a marker of domain-general processing efficiency in early numerical development. Non-symbolic quantity reproduction, by contrast, was largely accounted for by age and RAN, with no executive component contributing robustly after correction for multiple comparisons.

These findings point to a varied rather than uniform link between executive functions and early numerical skills, with separate executive elements supporting different numerical abilities. In practical terms, the results imply that early educational efforts for numerical growth should avoid treating executive functions as a single block. Programs designed to improve procedural counting may gain from tasks that draw on verbal working memory, while efforts focused on approximate quantity estimation could benefit from activities requiring suppression of perceptual distractors. The cross-cutting role of rapid naming also suggests that strengthening automatic retrieval may help across numerical areas.

## Figures and Tables

**Table 1:** Demographic characteristics of the sample.

	*N*	%	Mean Age (SD)	IQR
Total N	316	100	59.85 (9.66)	15.00
Females	158	50	60.03 (9.07)	14.75
Males	158	50	59.66 (10.25)	16.50

**Table 2:** Descriptive Statistics.

	Magic Box Task (Inhibitory Control)	Sentence Repetition (VSTM)	Animal House (VSWM)	What’s in Common Task (Set-Shifting)	ANS (Non-Symbolic Quantity Comparison)	Golden Tokens (Non-Symbolic Quantity Reproduction)	Ball Jar (Procedural Cardinality)	Receptive Language Task (Syntactic Comprehension)	RAN (Rapid Automatized Naming)
N	298	310	304	302	293	284	274	306	268
Missing	18	6	12	14	23	32	42	10	48
Mean	5.513	5.929	29.829	14.490	8.512	2.894	8.865	5.644	46.642
Median	6	6	31	15	9	3	12	6	47
Standard deviation	2.273	2.111	5.573	2.919	1.776	1.981	3.950	1.950	13.801
IQR	3.000	4.000	7.000	4.000	2.000	4.000	6.000	3.000	19.000
Minimum	0	0	3	3	0	0	0	0	5
Maximum	8	12	41	18	10	6	12	9	95
Skewness	−0.745	−0.199	−1.306	−1.225	−1.777	0.123	−0.858	−0.703	0.045
Std. error skewness	0.141	0.138	0.140	0.140	0.142	0.145	0.147	0.139	0.149
Kurtosis	−0.437	0.536	2.937	1.691	3.795	−1.264	−0.727	0.244	0.172
Std. error kurtosis	0.281	0.276	0.279	0.280	0.284	0.288	0.293	0.278	0.297
Shapiro–Wilk W	0.891	0.943	0.919	0.895	0.785	0.915	0.769	0.939	0.997
Shapiro–Wilk *p*	<.001	<.001	<.001	<.001	<.001	<.001	<.001	<.001	.953

**Table 3:** Spearman correlations among executive function components, domain-specific numerical precursors and cognitive variables.

Variable	1	2	3	4	5	6	7	8	9	10
1. Age in months	—									
2. Inhibitory control	0.266 ***	—								
3. VSTM	0.407 ***	0.256 ***	—							
4. VSWM	0.581 ***	0.185 **	0.275 ***	—						
5. Set-shifting	0.425 ***	0.186 **	0.269 ***	.294 ***	—					
6. Receptive language	0.485 ***	0.233 ***	0.296 ***	0.342 ***	0.284 ***	—				
7. RAN	0.561 ***	0.214 ***	0.347 ***	0.511 ***	0.321 ***	0.398 ***	—			
8. ANS	0.493 ***	0.249 ***	0.334 ***	0.343 ***	0.256 ***	0.280 ***	0.443 ***	—		
9. Procedural cardinality	0.578 ***	0.292 ***	0.433 ***	0.445 ***	0.250 ***	0.357 ***	0.477 ***	0.436 ***	—	
10. Non-symbolic quantity reproduction	0.542 ***	0.208 ***	0.335 ***	0.387 ***	0.365 ***	0.249 ***	0.466 ***	0.375 ***	0.449 ***	—

Note. N = 316. Pooled Spearman correlations computed on 50 imputed datasets. VSTM = Verbal Short-Term Memory; VSWM = Visuospatial Working Memory; RAN = Rapid Automatized Naming; ANS = Approximate Number System (non-symbolic quantity comparison). ** *p* < .01. *** *p* < .001.

**Table 4:** Hierarchical regression models: final coefficients (B, 95% CI, β) and step-wise incremental variance (multiple imputation vs. complete case).

Predictor	Procedural Cardinality	Non-Symbolic Quantity Reproduction	ANS
	B (95% CI) β	B (95% CI) β	B (95% CI) β
Age (months)	0.132 *** (0.077, 0.186) β = 0.317	0.074 *** (0.045, 0.103) β = 0.360	0.041 ** (0.015, 0.067) β = 0.219
Receptive language	0.040 (−0.194, 0.274) β = 0.020	−0.078 (−0.195, 0.039) β = −0.077	−0.045 (−0.153, 0.063) β = −0.049
RAN	0.047 * (0.011, 0.083) β = 0.169	0.025 ** (0.007, 0.043) β = 0.182	0.031 ** (0.013, 0.050) β = 0.252
VSTM	0.388 *** (0.172, 0.603) β = 0.205	0.073 (−0.037, 0.184) β = 0.078	0.097 (−0.005, 0.198) β = 0.115
VSWM	0.068 (−0.014, 0.150) β = 0.094	0.017 (−0.026, 0.061) β = 0.048	0.012 (−0.027, 0.050) β = 0.037
Inhibitory control	0.191 (−0.003, 0.385) β = 0.109	0.033 (−0.063, 0.129) β = 0.038	0.150 *** (0.062, 0.238) β = 0.191
Set-shifting	−0.038 (−0.179, 0.103) β = −0.028	0.080 * (0.009, 0.151) β = 0.118	0.018 (−0.048, 0.085) β = 0.030
Step-wise variance explained			
	MI|CC	MI|CC	MI|CC
Step 1: Age	R^2^ = 0.343|0.292	R^2^ = 0.293|0.264	R^2^ = 0.224|0.228
Step 2: + Receptive language, RAN	ΔR^2^ = 0.044 ***|0.037 **	ΔR^2^ = 0.033 **|0.032 **	ΔR^2^ = 0.062 ***|0.049 ***
Step 3: + VSTM, VSWM	ΔR^2^ = 0.044 ***|0.030 **	ΔR^2^ = 0.008|0.003	ΔR^2^ = 0.018 *|0.005
Step 4: + Inhibitory control	ΔR^2^ = 0.011|0.007	ΔR^2^ = 0.002|0.000	ΔR^2^ = 0.033 ***|0.021 **
Step 5: + Set-shifting	ΔR^2^ = 0.001|0.004	ΔR^2^ = 0.012 *|0.008	ΔR^2^ = 0.001|0.000
**Total R^2^ (final model)**	**0.443|0.369**	**0.349|0.307**	**0.337|0.303**

Note. N = 316 for multiple imputation (MI) analyses; complete-case (CC) sample sizes: procedural cardinality N = 223; non-symbolic quantity reproduction N = 234; ANS N = 238. Pooled coefficients (B), 95% confidence intervals, and standardized coefficients (β) are reported for the multiple-imputation final models. Standardized coefficients allow direct comparison of predictor magnitudes across models. Step-wise variance explained is reported for both MI and CC analyses to facilitate sensitivity assessment. VSTM = Verbal Short-Term Memory; VSWM = Visuospatial Working Memory; RAN = Rapid Automatized Naming; ANS = Approximate Number System (non-symbolic quantity comparison). * *p* < .05. ** *p* < .01. *** *p* < .001.

**Table 5:** Moderation analyses of the working memory–numeracy relationship by age.

Outcome	Interaction Term	B (SE)	95% CI	*p*	*p* Bonferroni
Procedural cardinality	Age × VSTM	−0.017 (0.008)	[−0.033, −0.001]	.044	.262
	Age × VSWM	−0.002 (0.003)	[−0.009, 0.004]	.448	1.000
Non-symbolic quantity reproduction	Age × VSTM	0.005 (0.005)	[−0.005, 0.015]	.318	1.000
	Age × VSWM	0.001 (0.002)	[−0.002, 0.005]	.490	1.000
ANS	Age × VSTM	−0.005 (0.005)	[−0.016, 0.006]	.350	1.000
	Age × VSWM	−0.001 (0.002)	[−0.004, 0.002]	.504	1.000

Note. N = 316. Pooled unstandardized regression coefficients from three general linear models (one per outcome) estimated on the imputed datasets, with age, VSTM, VSWM, and their two-way interactions as predictors. All continuous predictors were mean-centered within each imputed dataset. Robust HC1 standard errors were used. Bonferroni correction was applied across the six interaction terms tested (adjusted α = 0.0083). VSTM = Verbal Short-Term Memory; VSWM = Visuospatial Working Memory; ANS = Approximate Number System. None of the six interactions survived correction.

**Table 6:** Sensitivity analysis: comparison of coefficients from multiple-imputation and complete-case hierarchical regression models.

Outcome	Predictor	Multiple Imputation (MI)			Complete Case (CC)			Assessment
		B	β	*p*	B	β	*p*	
Procedural cardinality	Age	0.132	0.317	<.001	0.137	0.354	<.001	Robust
	RAN	0.047	0.169	.011	0.046	0.175	.008	Robust
	VSTM	0.388	0.205	<.001	0.336	0.186	.003	Robust
	Inhibitory control	0.191	0.109	.054	0.157	0.090	.113	Not confirmed
Non-symbolic quantity reproduction	Age	0.074	0.360	<.001	0.078	0.371	<.001	Robust
	RAN	0.025	0.182	.006	0.027	0.185	.007	Robust
	Set-shifting	0.080	0.118	.028	0.064	0.096	.112	Not confirmed
ANS	Age	0.041	0.219	.002	0.051	0.321	<.001	Robust
	RAN	0.031	0.252	.001	0.026	0.246	<.001	Robust
	Inhibitory control	0.150	0.191	<.001	0.105	0.152	.009	Robust

Note. N = 316 for multiple imputation (MI) analyses; complete-case (CC) sample sizes: procedural cardinality N = 223; non-symbolic quantity reproduction N = 234; ANS N = 238. Only predictors that emerged as significant or nominal in the MI analyses are shown; predictors that were non-significant in both MI and CC analyses are omitted for brevity. B = unstandardized pooled regression coefficient; β = standardized coefficient. Robust = effect significant at the individual-coefficient level in both MI and CC analyses. Not confirmed = effect nominally significant or borderline in MI but not confirmed in the CC analysis. Absolute β differences between MI and CC for the seven robust effects were all below |Δβ| = 0.11 (median |Δβ| = 0.020). VSTM = Verbal Short-Term Memory; RAN = Rapid Automatized Naming; ANS = Approximate Number System (non-symbolic quantity comparison).

## Data Availability

The original contributions presented in this study are included in the article. Further inquiries can be directed to the corresponding author.

## Abbreviations

The following abbreviations are used in this manuscript:

ANS	Approximate Number System
CC	Complete Case
DAP-T	Digital Assessment of Prerequisites—Touchscreen
EF	Executive Function
EFs	Executive Functions
HC1	Heteroskedasticity-Consistent (standard errors, type 1)
IQR	Interquartile Range
MCAR	Missing Completely At Random
MI	Multiple Imputation
MICE	Multiple Imputation by Chained Equations
RAN	Rapid Automatized Naming
SES	Socioeconomic Status
VSTM	Verbal Short-Term Memory
VSWM	Visuospatial Working Memory
